# *Clostridium butyricum* MIYAIRI 588 Modifies Bacterial Composition under Antibiotic-Induced Dysbiosis for the Activation of Interactions via Lipid Metabolism between the Gut Microbiome and the Host

**DOI:** 10.3390/biomedicines9081065

**Published:** 2021-08-22

**Authors:** Tadashi Ariyoshi, Mao Hagihara, Susumu Tomono, Shuhei Eguchi, Ayaka Minemura, Daiki Miura, Kentaro Oka, Motomichi Takahashi, Yuka Yamagishi, Hiroshige Mikamo

**Affiliations:** 1 Department of Clinical Infectious Diseases, Aichi Medical University Graduate School of Medicine, Nagakute 480-1195, Japan; t.ariyoshi@miyarisan.com (T.A.); hagimao@aichi-med-u.ac.jp (M.H.); k.oka@miyarisan.com (K.O.); motomichi.takahashi@miyarisan.com (M.T.); y.yamagishi@mac.com (Y.Y.); 2 Miyarisan Pharmaceutical Co., Ltd., R&D Division, Saitama 331-0804, Japan; s.eguchi@miyarisan.com (S.E.); a.minemura@miyarisan.com (A.M.); da.miura@miyarisan.com (D.M.); 3 Department of Molecular Epidemiology and Biomedical Sciences, Aichi Medical University, Nagakute 480-1195, Japan; 4 Department of Microbiology and Immunology, Aichi Medical University Graduate School of Medicine, Nagakute 480-1195, Japan; tomono@aichi-med-u.ac.jp

**Keywords:** *Clostridium butyricum*, protectin D1, G-protein coupled receptor 120

## Abstract

The gut microbiome is closely related to gut metabolic functions, and the gut microbiome and host metabolic functions affect each other. *Clostridium butyricum* MIYAIRI 588 (CBM 588) upregulates protectin D1 production in host colon tissue following G protein-coupled receptor (GPR) 120 activation to protect gut epithelial cells under antibiotic-induced dysbiosis. However, how CBM 588 enhances polyunsaturated fatty acid (PUFA) metabolites remains unclear. Therefore, we focused on the metabolic function alterations of the gut microbiome after CBM 588 and protectin D1 administration to reveal the interaction between the host and gut microbiome through lipid metabolism during antibiotic-induced dysbiosis. Consequently, CBM 588 modified gut microbiome and increased the butyric acid and oleic acid content. These lipid metabolic modifications induced GPR activation, which is a trigger of ERK 1/2 signaling and directed differentiation of downstream immune cells in the host colon tissue. Moreover, endogenous protectin D1 modified the gut microbiome, similar to CBM 588. This is the first study to report that CBM 588 influences the interrelationship between colon tissue and the gut microbiome through lipid metabolism. These findings provide insights into the mechanisms of prevention and recovery from inflammation and the improvement of host metabolism by CBM 588.

## 1. Introduction

The gut microbiome contributes to the maintenance of homeostasis in host metabolism [1,2]. When the gut microbiome is disturbed, metabolites also fluctuate significantly [3]. Disturbance of this complex system is associated with diseases such as inflammatory bowel diseases (IBDs), which present with chronic persistent inflammation of the digestive tract [4,5]. Moreover, not only gastrointestinal mucosa-related diseases but also insulin resistance, obesity, and non-alcoholic fatty liver disease (NASH) have revealed that gut dysbiosis is one of the causes of abnormal host metabolism due to a decrease in beneficial bacteria [6,7,8]. Alterations in gut immune cell constitution and lipid metabolism are related to antibiotic-induced dysbiosis and exacerbate gut inflammation, mucosa, and epithelial damage in the colon of mice [9,10]. Currently, to develop a treatment method for dysbiosis-induced gut inflammation, research on live biotherapeutic products (LBPs) has been enthusiastically carried out [11,12].

*Clostridium butyricum* MIYAIRI 588 (CBM 588), a gram-positive obligate anaerobic bacillus, is an LBP that has been used as an anti-diarrheal agent in Japan [13]. CBM 588 not only suppresses diarrhea, but also has an effect against diseases that are caused by inflammation and dysbiosis, such as the prevention of ulcerative colitis, and has a positive impact on therapeutic efficacy in anti-tumor immune response [14,15].

Basic research results show that CBM 588 suppresses gut inflammation caused by antibiotic treatment, and host metabolic alteration induced by CBM 588 administration results in the upregulation of anti-inflammatory lipid mediators [9,10]. In our previous study, CBM 588 administration induced the anti-inflammatory lipid mediator, protectin D1, through activation of 15-lipoxygenase (LOX) by increasing G-protein coupled receptor (GPR) 120 and interleukine-4 (IL-4)-producing CD4^+^ cell population in colon tissue under antibiotic-induced dysbiosis [9].

Taking these results into account, we posed two questions. First, how CBM 588 increases polyunsaturated fatty acid (PUFA) metabolites in colon tissue needs to be elucidated. Second, we need to clarify whether CBM 588-induced lipid metabolites in the host colon tissue affect the composition changes of the gut microbiome during antibiotic-induced gut dysbiosis. Therefore, we focused on the alteration of gut microbiome metabolic functions after CBM 588 or protection D1 administration to reveal the interaction between the host and gut microbiome through lipid metabolism when the host is undergoing antibiotic-induced dysbiosis.

In this study, we investigated the composition of the gut microbiome and lipid metabolism during antibiotic-induced dysbiosis. In addition, we investigated the gene and protein expression levels related to lipid metabolism and immune cell signaling in host colon tissue under the same conditions to provide insights into the prevention and treatment of inflammation and improved host metabolism with CBM 588.

## 2. Materials and Methods

### 2.1. Reagents

CBM 588 powder was obtained from Miyarisan Pharmaceutical Co., Ltd. (Tokyo, Japan). The powder consisted of 3.3 × 10^10^ colony-forming units (cfu)/g (Lot No. 09GW) of viable spores of CBM 588. The powder was used for all in vivo studies. Clindamycin (Dalacin^®^ S Injection; CLDM) was purchased from Pfizer Japan Inc. (Tokyo, Japan). Protectin D1 (10(R), 17(S)-dihydroxy-4Z,7Z,11E,13Z,15E,19Z-docosahexaenoic acid) was purchased from Cayman USA Inc. (Ann Arbor, MI, USA). Immediately before each in vivo experiment, the CBM 588 powder was weighed and reconstituted to the required amount with phosphate-buffered saline (1 × PBS, pH 7.4). CLDM and protectin D1 were further diluted in PBS to achieve the desired concentrations. Both suspensions were refrigerated during storage and discarded 12 h after reconstitution.

### 2.2. Mouse Preparation and Housing

Specific-pathogen-free, female ICR mice (8–9 weeks old) were obtained from Charles River Laboratories Japan, Inc. (Kanagawa, Japan) (body weight is approximately 30 g) and utilized throughout this experiment. The mice were maintained according to the National Research Council recommendations. They were provided food and water ad libitum. The study was reviewed and approved by the Ethics Committee of Aichi Medical University (approval date: 13 July 2020, approval code: #2020-29).

### 2.3. Administration of Medicines

For the in vivo study, 25 female ICR mice were divided into five groups (*n* = 5): (I) control group, (II) CLDM administration group, (III) CBM 588 administration group, (IV) CBM 588 + CLDM combination group, and (V) protectin D1 + CLDM combination group. CBM 588 powder was administered by oral gavage at 500 mg/kg/day (3.4 × 10^8^ cfu/g/mouse/day). CLDM was administered by oral gavage at a dose of 40 mg/kg/day. The CBM 588 powder was dissolved in PBS and mixed. The relevant medicines were administered to the groups of mice, twice daily, at 10 a.m. and 4 p.m., using 1/2 doses each time for 4 days and kept for another 4 days [10]. Protectin D1 was administered by intraperitoneal injection (IP) at 0.3 µg/100 µL/mouse /day [16].

### 2.4. DNA Extraction and Purification

Fecal samples from each mouse were analyzed by sequencing the 16S rRNA gene V3–V4 regions. DNA extraction and sequencing of 16S rRNA-encoding gene amplicons were conducted as previously described [10]. Briefly, fecal pellets were suspended in 10 mM Tris-HCl and 10 mM EDTA buffer (pH 8.0) and incubated with lysozyme (Sigma-Aldrich, St. Louis, MO, USA) (final concentration: 15 mg/mL) at 37 °C for 1 h. Purified achromopeptidase (FUJIFILM Wako Pure Chemical Co., Osaka, Japan) was added (final concentration: 2000 U/mL) and further incubated at 37 °C for another 30 min. Sodium dodecyl sulfate (FUJIFILM Wako Pure Chemical Co., final concentration: 1%) was then added to the cell suspension and mixed well. Subsequently, proteinase K (Takara Bio Inc., Shiga, Japan) was added (final concentration: 1 mg/mL) to the suspension, and the mixture was incubated at 55 °C for 1 h. DNA was isolated by using the phenol/chloroform extraction, followed by precipitation using 75% ethanol. Then, the supernatant was transferred and added an equal amount of isopropanol and 3 M sodium acetate (5% *w*/*v*). After centrifugation, the supernatant was washed with 75% ethanol and centrifuged to remove the supernatant. Further, 200 µL of TE and 1 µL of RNase were added and incubated at 37 °C for 1 h. Finally, the extracted DNA was purified using the High Pure PCR Template Preparation Kit (Roche Diagnostics, Mannheim, Germany). All procedures were performed according to the manufacturer’s instructions.

### 2.5. Gut Microbiome Analysis

Meta 16S rRNA gene sequencing PCR was performed using Ex Taq Hot Start (Takara Bio) and the Illumina forward primer 5′-AATGATACGGCGACCACCGAGATCTACAC (adaptor sequence) +barcode (eight bases) + ACACTCTTTCCCTACACGACGCTCTTCCGATCT (sequence primer) + CCTACGGGNGGCWGCAG-3′ (341F) and the Illumina reverse primer 5′-CAAGCAGAAGACGGCATACGAGAT (adaptor sequence) + barcode (eight bases) + GTGACTGGAGTTCAGACGTGTGCTCTTCCGATCT (sequence primer) + GACTACHVGGGTATCTAATCC-3′ (805R) to the hypervariable V3–V4 region of the 16S rRNA gene. Amplicons generated from each sample were subsequently purified using SPRISelect (Beckman Coulter, Brea, CA, USA). DNA was quantified using a Quantus Fluorometer and ONEdsDNA System (Promega, Madison, WI, USA). Mixed samples were prepared by pooling approximately equal amounts of amplified DNA and sequenced using MiSeq Reagent Kit V3 (600 cycle) and MiSeq sequencer (Illumina, San Diego, CA, USA), according to the manufacturer’s instructions. The 16S rRNA gene sequence data generated by the MiSeq sequencer (Illumina) were processed using quantitative insights into microbial ecology 2 (QIIME 2 October 2019) pipeline [17]. Raw sequence data were demultiplexed and quality filtered using the q2-demux plugin followed by denoising with DADA2 [18]. All amplicon sequence variants (ASVs) were aligned with MAFFT [19] and used to construct a phylogeny using FastTree2 [20]. Taxonomy was assigned to ASVs using the q2-feature-classifier [21] classify-sklearn naïve Bayes taxonomy classifier against SILVA 138.1.

### 2.6. Alpha (α)-and Beta (β)-Diversity Analysis

Within-community diversity (α-diversity) was calculated using QIIME2. An α-rarefaction was generated using a Chao 1 estimator of species richness with 10 sampling repetitions at each sampling depth [22]. An even depth of ~20527 sequences per sample was used for the calculation of richness and diversity indices. To compare microbial composition between samples, β-diversity was measured by calculating the weighted UniFrac distances using QIIME2 default scripts [23]. Principal coordinate analysis (PCoA) was applied to the resulting distance matrices to generate two-dimensional plots. Each colored point represents a fecal sample obtained from one mouse coloring according to different treatments (control, CLDM administration group, CBM 588 administration group, CBM 588 + CLDM administration group, and protectin D1 + CLDM administration group).

### 2.7. Predictive Functional Profiling of Gut Microbial Communities

To gain more insight into the metagenomics-based function of the microbiome in each group of mice, the Phylogenetic Investigation of Communities by Reconstruction of Unobserved States (PICRUSt) v2.4.1 [24] was used to obtain relative Kyoto Encyclopedia of Genes and Genomes (KEGG) pathway abundance information derived from metagenomics data. The predicted data were collapsed into hierarchical categories, and the relative abundances of gut metabolic functions were calculated and graphed using R software Version 4.1.0. The heatmap was generated by hierarchical clustering of the relative abundance of metabolic pathways with Z-score normalization.

### 2.8. Linear Discriminant Analysis (LDA) Effect Size (LEfSe)

A LEfSe approach was used to identify bacterial taxa that were significantly differentially abundant between each treatment group [25]. Only taxa with a > 3 log 10 LDA score were considered significantly enriched at a *p* value < 0.05.

### 2.9. Fecal Sample Preparation for Long-Chain Fatty Acid Metabolome Analysis

Feces were sampled from the mouse colon and immediately cryopreserved (stored at −80 °C). In addition, fecal samples were subjected to the following operations on ice: Samples were lyophilized, weighed for 10 mg, 500 µL methanol was added, and the mixture was vortexed for 1 min. These samples were centrifuged (15,000× *g*, 3 min), dried in vacuo, dissolved in 100 µL methanol, and vortexed for 1 min. The samples were centrifuged (15,000× *g*, 3 min) and the supernatants were collected. The supernatants were used for liquid chromatography–tandem mass spectrometry (LC-MS/MS). The culture supernatant of CBM 588, which is described later in Section 2.12, was also treated using the same method.

### 2.10. Targeted Long Chain Fatty Acid Metabolites Analysis Using LC-MS/MS

The orbitrap LC-MS/MS analyses were performed on a Vanquish H system (Thermo Fisher Scientific, Marietta, OH, USA ) using an Acclaim RSLC120 C18 (2.2 µm 2.1 i.d. × 150 mm, Thermo Fisher Scientific) and Q Exactive (Thermo Fisher Scientific) with an electrospray ionization device. Detailed conditions and experimental methods are described in the supplemental materials (the linear gradient conditions, MS conditions, and ionization conditions are listed in Tables S1–S3).

### 2.11. Organic Acid Measurement Using High Performance Liquid Chromatography (HPLC)

Feces were sampled from the mouse colon and immediately cryopreserved (stored at −80 °C). Organic acids (acetic acid, propionic acid, n-butyric acid, iso-valeric acid, succinic acid, and lactic acid) in feces were measured using high-performance liquid chromatography (Prominence, SHIMADZU, Kyoto, Japan). Detailed conditions and experimental methods are described in the supplemental materials.

### 2.12. CBM 588 Culture with Linoleic Acid

As controls, CBM 588 non-inoculated Gifu anaerobic medium (GAM) broth supplemented without (I) or with 50 µM linoleic acid (II) were also treated under the same conditions as mentioned later. CBM 588 powder was suspended in 1 × PBS to 1 × 10^8^ cfu/mL in a 1.5 mL microcentrifuge tube. One hundred microliters of CBM 588 suspensions were added to 900 µL of fresh GAM broth (Nissui Seiyaku Co., Ltd., Tokyo, Japan) supplemented without (III) or with 50 µM of linoleic acid (Sigma Aldrich, St. Louis, MO, USA) (IV) and cultured at 37 °C under anaerobic conditions for 24 h. After culturing, the suspension was centrifuged at 10,000× *g* for 5 min at 4 °C and the supernatant was filtered through a 0.45 µm filter (Merck Millipore). These samples were triplicated, and the long-chain fatty acid metabolites were measured by LC-MS/MS using the same method as described in Section 2.9 and Section 2.10.

### 2.13. Cell Culture and Treatment

To investigate ligands that highly activate GPR120, which is known to activate MAP kinase ERK 1/2 in different cellular systems, we evaluated cell activation with the measurement of ERK 1/2. Then, we followed previous study methods [26,27] with some modifications. Detailed methods are described in the supplemental materials.

### 2.14. RNA Isolation and cDNA Preparation

As previously described [10], mouse colons (30 mg) were homogenized, and total RNA was isolated by using an RNA isolation kit (MACHEREY-NAGEL, Düren, Germany) according to the manufacturer’s protocol. Subsequently, to prepare complementary DNA (cDNA), a high-capacity RNA to cDNA kit (Thermo Fisher Scientific) was used in accordance with the manufacturer’s instructions. Total RNA solution (9 μL) was mixed with 2× RT Buffer mix and 20× RT Enzyme Mix to obtain a final volume of 20 μL. The mixture was incubated at 37 °C for 60 min, heated to 95 °C for 5 min, and held at 4 °C. For convenience, incubation was performed in a thermal cycler. The cDNA was either immediately used in real-time PCR applications or placed for long-term storage in a freezer (−20 °C).

### 2.15. Quantitative Real-Time Polymerase Chain Reaction (RT-PCR)

PowerUp^TM^ SYBR Green PCR Master Mix (Applied Biosystems, Foster City, CA, USA) was used to conduct quantitative RT-PCR according to the manufacturer’s protocol. PCR reactions were performed with reaction mixture containing of cDNA (2 μL), PowerUpTM SYBR Green PCR Master Mix (5 μL), 10 μM forward primer (0.5 μL), 10 μM reverse primer (0.5 μL), and nuclease-free water (2 μL) (total volume: 10 μL). Primer sequences are listed in Table S4. The following RT-PCR protocol was used: (i) Tm ≥ 60 °C, 50 °C (2 min), 95 °C (2 min), and 40 cycles of denaturation at 95 °C (15 s), annealing at 60 °C (1 min), (ii) Tm < 60 °C: 50 °C (2 min), 95 °C (2 min), with 40 cycles of denaturation at 95 °C (15 s), annealing at 60 °C (15 s), and extension at 72 °C (1 min). For relative quantitation, we used β-actin as an endogenous reference to compare the amount of normalized target and the ΔΔCt method to analyze the data. Relative RNA expression levels were normalized to that of the control group (represented as RQ).

### 2.16. Proteins Related to Lipid Metabolism

Colon tissue samples were homogenized in RIPA buffer (Nacalai Tesque, Kyoto, Japan). After sonication (10 s), the suspension was centrifuged at 10,000× *g* for 20 min at 4 °C. The supernatant was collected as a sample. To investigate alterations in fatty acid metabolism and signaling factors of immune cells, GPR41 (BioLegend, San Diego, CA, USA), GPR109a (MyBioSource, Inc., San Diego, CA, USA), GPR120 (BioLegend), FADS1 (Bio-connect, Huissen, Netherlands), ELOVL5 (Abbexa, Cambridge, UK), and GATA3 (MyBioSource) in the colon tissue supernatants were measured using commercially available mouse enzyme-linked immunosorbent assay (ELISA) kits. All procedures were performed according to the manufacturer’s instructions.

### 2.17. Statistics and Analysis

For quantitative data, except for the CBM 588 culture supernatant samples (*n* = 3), all results are shown as the mean ± SD (*n* = 5). One-way ANOVA with Tukey’s test was applied to compare the total number of genes in the extracted pathway by PICRUSt2 analysis, LC-MS/MS signal intensity, gene expression level (RT-PCR), and protein expression level (ELISA). A Kruskal–Wallis test was employed for the statistical analysis of α-diversity and LEfSe analysis (*p* < 0.05). β-Diversity *p*-values were calculated using PERMANOVA. Samples were clustered according to the treatment status of the mice (*p* < 0.05).

## 3. Results

### 3.1. CBM 588 and Protectin D1 Modulated Gut Microbiome Composition under Antimicrobial Administration

**Figure 1:**
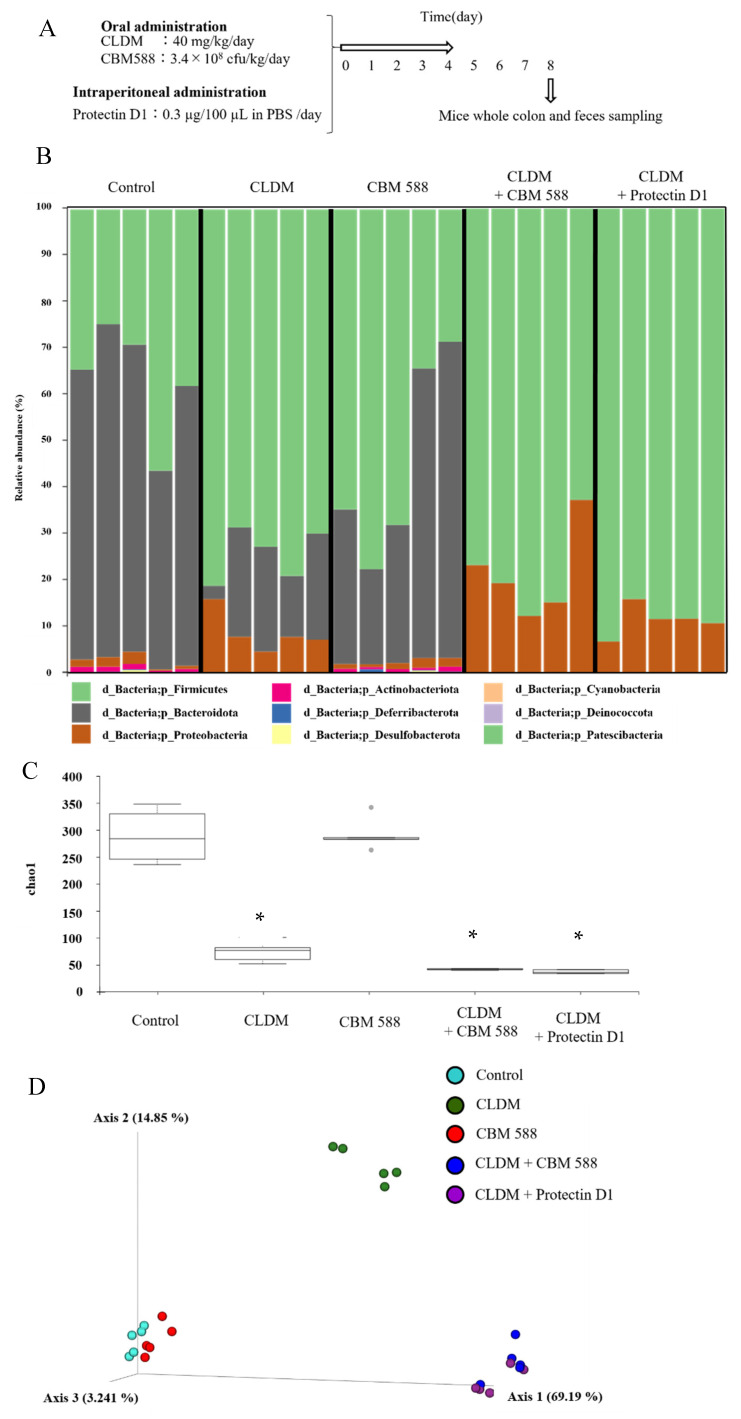

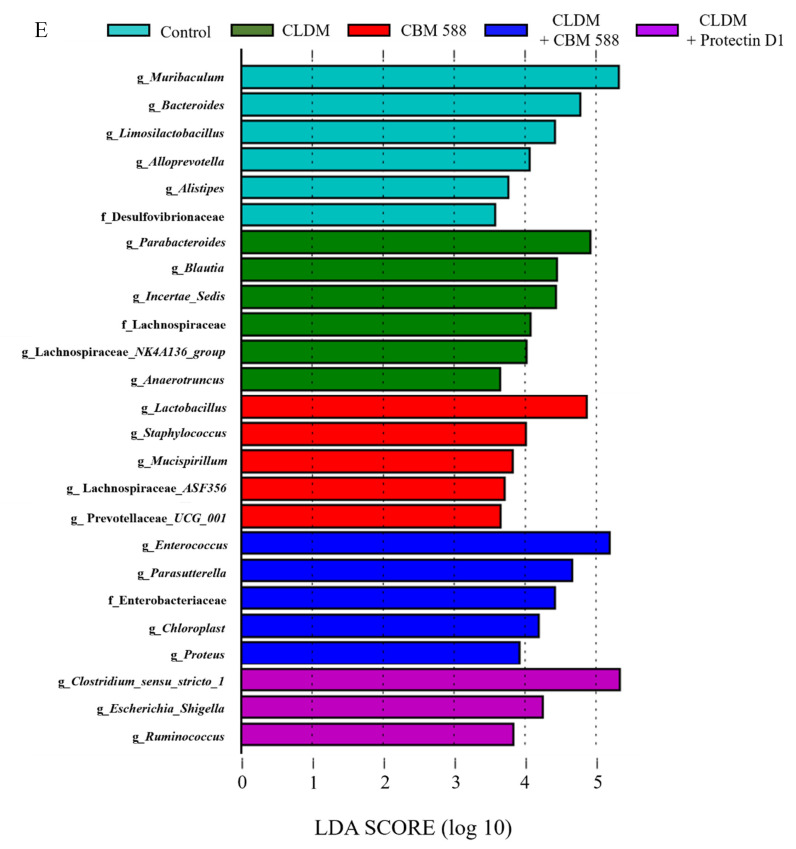
CBM 588 and protectin D1 both modulate gut microbiome following antimicrobial administration. (**A**) Experimental design of in vivo study with 9-week-old ICR mice. Mice were divided into groups as follows and received CBM 588 and/or CLDM via sonde, protectin D1 by intraperitoneal administration for 4 d: control group (Control), CLDM administration group (CLDM), CBM 588 administration group (CBM 588), CBM 588 combination group (CBM588 + CLDM) and protectin D1 combination group (protectin D1 + CLDM). (**B**) Bacterial compositions in different experimental groups at the phylum level. The vertical axis shows relative abundance (%) per the whole (*n* = 5 in each group). (**C**) The mean Chao1 index between each group. The vertical axis shows Chao 1 index. The box and whiskers represent the smallest and largest values, with the median in the center of each box (*n* = 5). The probability was considered statistically significant at *p* < 0.05. *: *p* < 0.05 compared with control. (**D**) Principal coordinates analysis (PCoA) derived from unweighted UniFrac distances (*n* = 5). (**E**) LEfSe analysis of the gut microbiome composition. Histogram of the LDA scores (log 10) > 4.0 and *p* < 0.05 reveals the most differentially abundant taxa among different reproductive stages. g_ means genus, f_ means family (*n* = 5).

To elucidate the effects of CBM 588 and protectin D1 on the gut microbiome following antibiotic administration, we administered CLDM, CBM 588, and/or protectin D1 to ICR mice for 4 d (Figure 1A), and investigated the gut microbiome using 16S rRNA gene sequencing. The gut microbiome at the phylum level is shown in Figure 1B. A high abundance of Bacteroidetes (Bacteroidota) was observed in the control and CBM 588-only administration groups. In contrast, the other three groups treated with clindamycin had a lower abundance of Bacteroidetes (Bacteroidota) and an increased abundance of Proteobacteria (Figure 1B). To compare the results of gut microbiome data, we determined α-diversity with Chao1 index (Figure 1C) and found that the mean Chao1 index was significantly lower in the groups that were administered CLDM only, CBM 588 + CLDM combination, and protectin D1 + CLDM combination groups than in the control group (*p* = 0.009, respectively). Additionally, β-diversity was evaluated by the unweighted UniFrac distance applied for PCoA at the ASV level and statistically analyzed by PERMANOVA. (Figure 1D). The CLDM-only administration group, CBM 588 + CLDM combination, and protectin D1 + CLDM combination group showed significant differences compared with the control group (*p* = 0.007, 0.014, and 0.011, respectively). However, the CBM 588 + CLDM combination group had significantly different gut microbiome compositions compared with the CLDM-only administration group (*p* = 0.028) and protectin D1 + CLDM combination group (*p* = 0.135). We then investigated gut microbiome changes at the genus level (Figure S1). The relative abundance of various families with percentages of sequences > 0.001% of the bacterial community was analyzed. To estimate more detailed group-specific bacteria, the LEfSe analyses revealed the enriched bacteria at the genus level with statistically significant changes in abundance among all groups (Figure 1E). The control group showed an increase in *Muribaculum*, *Bacteroides*, and *Limosilactobacillus*. The CLDM-only administration group showed an increase in the genera *Parabacteroides* and *Blautia*. Moreover, the CBM 588-only administration group showed a high abundance of *Lactobacillus*. The CBM 588 + CLDM-treated group showed an increase in *Enterococcus*, *Proteus*, and *Parasutterella*. Not only the CBM 588 + CLDM combination group, but also the protectin D1 + CLDM combination group increased the *Clostridium sensu stricto* 1.

### 3.2. Modified Gut Microbiome with CBM 588 and Protectin D1 Administrations Enhance Functional Genes Associated with Lipid Metabolism and Antioxidant Stress

**Figure 2:**
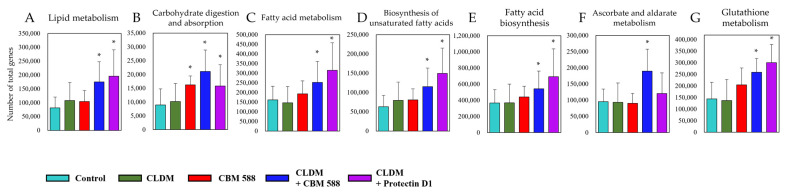
Gut microbiome modified with CBM 588 and protectin D1 enhances functional genes associated with lipid metabolism and antioxidant stress. Number of total genes in each pathway in lipid metabolism (**A**) carbohydrate digestion and absorption (**B**) fatty acid metabolism (**C**) biosynthesis of unsaturated fatty acids (**D**) fatty acid biosynthesis (**E**) ascorbate and aldarate metabolism (**F**) and glutathione metabolism (**G**). Statistical significance was set at *p* < 0.05. * *p* < 0.05, compared with control.

To elucidate the impact of CBM 588 and protectin D1 on the gut microbiome disrupted by CLDM administration, we investigated gut metabolic alterations in the resident gut microbiome (Figure S2). A total of 214 metabolic functions detected by the PICRUSt2 analysis from the gut microbiome data showed significantly different numbers of total genes in both treatment groups compared to the control group. The control group had a metabolic pathway pattern similar to that of the CBM 588 mono-administration group. Likewise, the protectin D1 + CLDM administration group and the CBM 588 + CLDM administration group also presented similar patterns of metabolic pathway activities. Of note, the CBM588 + CLDM combination group and protectin D1 + CLDM combination group showed significantly higher number of total genes in lipid metabolism (A), carbohydrate digestion and absorption (B), fatty acid metabolism (C), biosynthesis of unsaturated fatty acids (D) and fatty acid biosynthesis (E), ascorbate and aldarate metabolism (F), and glutathione metabolism (G) than the control group (Figure 2). The (A) to (E) pathways are related to the metabolism of unsaturated fatty acids and organic acids. The (F) and (G) pathways are related to anti-oxidative stress metabolism.

### 3.3. CBM 588 and Protectin D1 Administrations Result in the Enhancement of the Conversions from Linoleic Acid to Oleic Acid via CLAs and Upregulate the Productions of Butyric Acid and EPA-Related Metabolites in Gut Microbiome

**Figure 3:**
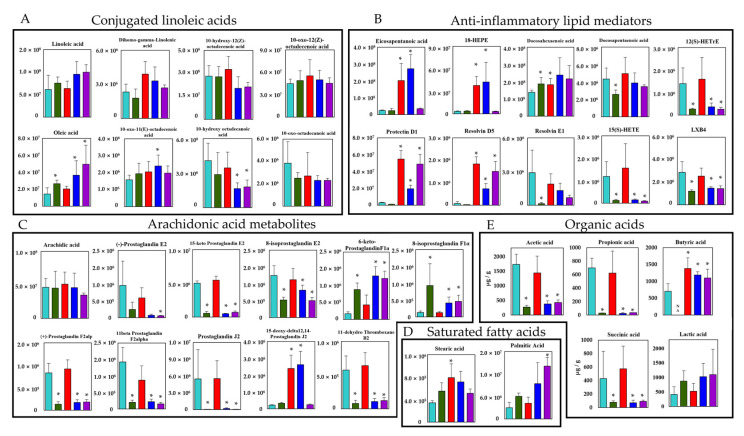
Verification of the butyric acid and EPA-related metabolites inducing efficacy by CBM 588 based on targeted long-chain fatty acid metabolites profiles. Metabolic pathway for PUFAs and SCFAs in mouse colon contents. The signal intensities of metabolites were compared using the average values of each group. The vertical axis indicates the signal intensity. The vertical axis of the SCFAs is the concentration (µg/g). (**A**) Conjugated linoleic acid (**B**) Anti-inflammatory lipid mediators. (**C**) Arachidonic acid metabolites. (**D**) Saturated fatty acids. (**E**) Organic acids. Values represent mean ± SD (*n* = 5). Statistical significance was set at *p* < 0.05. * *p* < 0.05, compared with control. HEPE: hydroxyeicosapentaenoic acid, HETrE: hydroxyeicosatrienoic acids, HETE: hydroxyeicosatrienoic acid, LXB_4_: lipoxin B_4_.

Next, we performed targeted long-chain fatty acid metabolite analysis with the fecal samples. A total of 52 lipid metabolites, including structural isomers, were assigned with orbitrap LC-MS/MS, and 25 metabolites showed significantly different peak intensities compared with the control group (*p* < 0.05) (Figure 3A = 3/8, B = 10/10, C = 10/11, and D = 2/2). Of note, among the CLAs, which are converted products of linoleic acid, oleic acid was increased in the CBM 588 + CLDM combination administration group and the protectin D1 + CLDM combination administration group, while 10-hydroxy octadecanoic acid was decreased in both groups. On the other hand, co-administration of CBM 588 resulted in a large increase in EPA and EPA-related metabolites, such as 18-hydroxy eicosapentaenoic acid (HEPE), protectin D1, and resolvin D5, even under CLDM treatment (Figure 3B). However, among the metabolites of arachidonic acid, such as prostaglandins, except 15-deoxy-delta12,14-prostaglandin J_2_, 6-keto-prostaglandin F_1α_, and 8-isoprostaglandin F_1α_, were found to be remarkably reduced in the CLDM-administration groups (Figure 3C). Two saturated fatty acids were assigned to this targeted lipid metabolomics analysis. Among them, stearic acid was significantly increased in the CBM 588-only administration group. Palmitic acid was significantly increased in the protectin D1 + CLDM combination group (Figure 3E).

In addition, quantitative measurements using HPLC to detect organic acids revealed that succinic acid, acetic acid, and propionic acid were significantly decreased in the CLDM-administered groups. Butyric acid was not detected in the CLDM-only administration group. However, the concentration of butyric acid was significantly increased not only in the CBM 588-only administration group but also in the CBM 588 + CLDM combination administration group and protectin D1 + CLDM combination administration group, compared with the control group (Figure 3E).

### 3.4. CBM 588 Can Metabolize Linoleic Acid to CLAs

**Figure 4:**
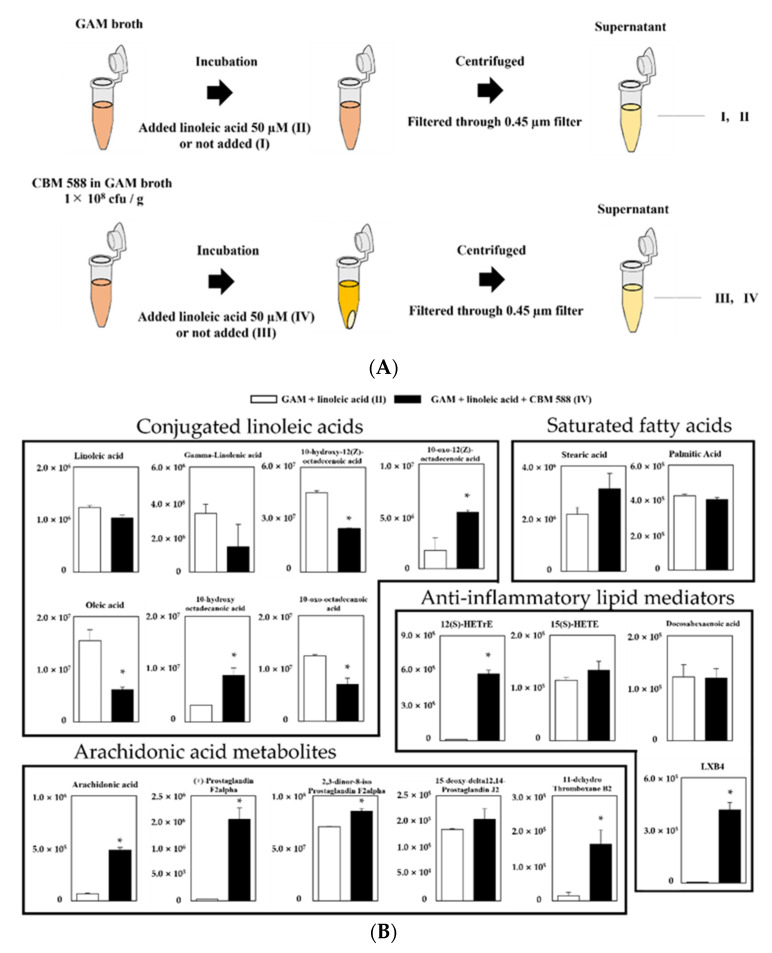
Evaluation of linoleic acid conversion during CBM 588 single-culture test based on target fatty acid metabolites profiles. (**A**) Experimental design of CBM 588 culturing. Culture broth were divided into four groups as follows, I: GAM broth, II: GAM-broth-containing linoleic acid 50 µM, III: supernatant after 24 h CBM 588 culture in GAM broth and IV: supernatant after 24 h CBM 588 culture in GAM-broth-containing linoleic acid 50 µM. (**B**) The metabolic pathway for the PUFAs in GAM broth culture medium (II and IV). The signal intensities of the metabolites were compared using the average value of each group. The vertical axis indicates the signal intensity. The values represent the mean ± SD (*n* = 5). A probability was considered statistically significant at *p* < 0.05. *: *p* < 0.05 when compared with control.

To investigate whether CBM 588 plays an important role in the conversion of linoleic acid to oleic acid under gut dysbiosis, we conducted a long-chain fatty-acid-targeted metabolomics analysis in several culture supernatants of CBM 588. Then, we prepared the following groups (I: GAM broth, II: GAM broth containing linoleic acid, III: supernatant after 24 h CBM 588 culture in GAM broth and IV: supernatant after 24 h CBM 588 culture in GAM broth containing linoleic acid) and investigated lipid metabolites in the broth with LC-MS/MS. A schematic culture diagram of each sample is shown in Figure 4A. Consequently, a total of 18 lipid metabolites were assigned to GAM-broth-containing linoleic acid (II) and supernatant after 24 h of CBM 588 culture in GAM-broth-containing linoleic acid (IV), while the signal intensity of the lipid metabolites was not detected in either GAM broth (I) or supernatant after 24 h of CBM 588 culture in GAM broth (III) (data not shown). Compared with GAM-broth-containing linoleic acid (II), 10-hydroxy octadecanoic acid and 10-oxo-12(Z)-octadecenoic acid increased in the supernatant after 24 h of CBM 588 culture in GAM-broth-containing linoleic acid (IV) (Figure 4B). Additionally, arachidonic acid metabolites and several anti-inflammatory lipid metabolites increased in the supernatant after 24 h of CBM 588 culture in GAM-broth-containing linoleic acid (IV).

### 3.5. CLAs and Other Metabolites CBM 588 Induced Activate MAP Kinase ERK 1/2

**Figure 5:**
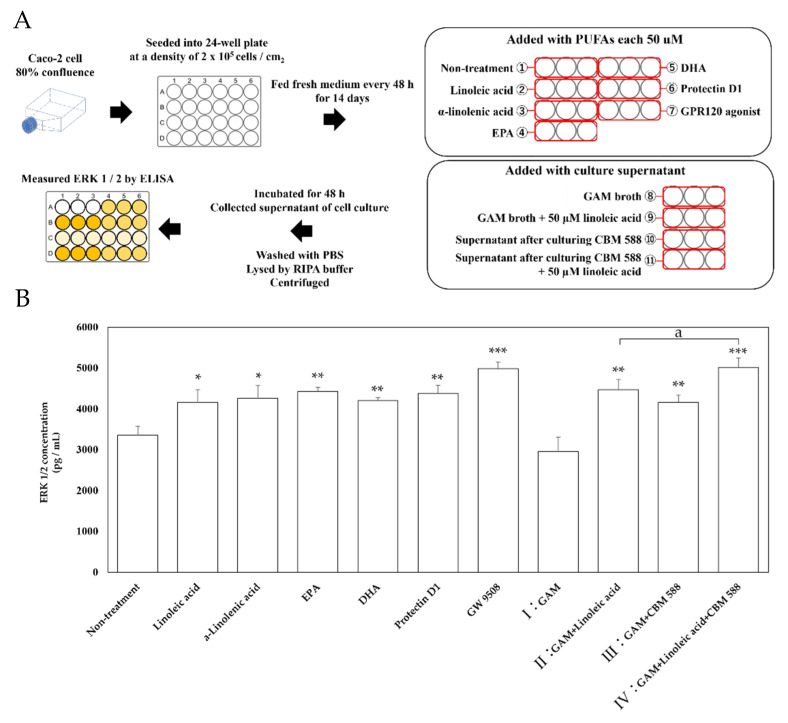
ERK 1/2 concentration in Caco-2 cells after ligand stimulations. (**A**) Experimental design of in vitro study with Caco-2 cells. Caco-2 cells were cultured at 37 °C in 75 cm^2^ tissue culture flasks to 80% confluence and then seeded into a 24-well micro plate at a density of 2 × 10^5^ cells / cm^2^. After 14 days of culture, the Caco-2 cells were treated with PUFAs (linoleic acid, α-linolenic acid, EPA and DHA each 50 µM), GPR120 agonist GW 9508, CBM 588 free culture medium (I: GAM broth and II: GAM broth supplemented with 50 µM linoleic acid, same samples as described in Section 2.12), and CBM 588 culture supernatant (III: supernatant after culturing CBM 588 in GAM broth and IV: supernatant after culturing CBM 588 in GAM broth supplemented with 50 µM linoleic acid, as described in Section 2.12), and the Caco-2 cells were incubated for 48 h. Protein expression levels of ERK 1/2 in the supernatants were measured with commercially available mouse ELISA kits. (**B**) Changes in ERK 1/2 activity by addition of fatty acid ligands, culture broth with or without linoleic acid, and CBM 588 to Caco-2 cells. The vertical axis shows concentration (pg/mL). The values represent the mean ± SD (*n* = 3, red circle mark). The calculated probability was considered statistically significant at *p* < 0.05. *: *p* < 0.05 compared with control. **: *p* < 0.01. ***: *p* < 0.001. a: *p* < 0.05 compared with II (GAM broth supplemented with 50 µM linoleic acid).

Next, we performed an in vitro ligand addition test study with Caco-2 cells, reported to express GPR120 [27], to investigate which metabolites induced by CBM 588 activate GPR120-mediated signaling. A schematic of the experiment is shown in Figure 5A. ERK 1/2 was used as an index of signal transduction from GPR120, and its expression level was evaluated. The addition of long-chain fatty acids, such as linoleic acid, α-linolenic acid, EPA, DHA, protectin D1, and GW9508 (positive control) resulted in the upregulation of ERK 1/2 protein concentration (Figure 5B). Additionally, ERK 1/2 protein expression was also upregulated when the supernatants of II (GAM-broth-containing linoleic acid) were added. Moreover, the supernatant of CBM 588 culture in GAM broth (III) showed similar ERK 1/2 expression levels with the addition of the supernatant of II (GAM-broth-containing linoleic acid). In addition, the ERK 1/2 concentration significantly increased in the supernatant of IV (after 24 h of culturing CBM 588 in GAM-broth-containing linoleic acid) rather than II. The supernatant of IV also showed the highest ERK 1/2 protein expression level, similar to the result of GW9508 (positive control).

### 3.6. The Gut Microbiome Altered by CBM 588 and/or Protectin D1 Upregulates Host Fatty Acid Receptors, Lipid Elongation Enzymes, and Immune Cell Signaling Factors

**Figure 6:**
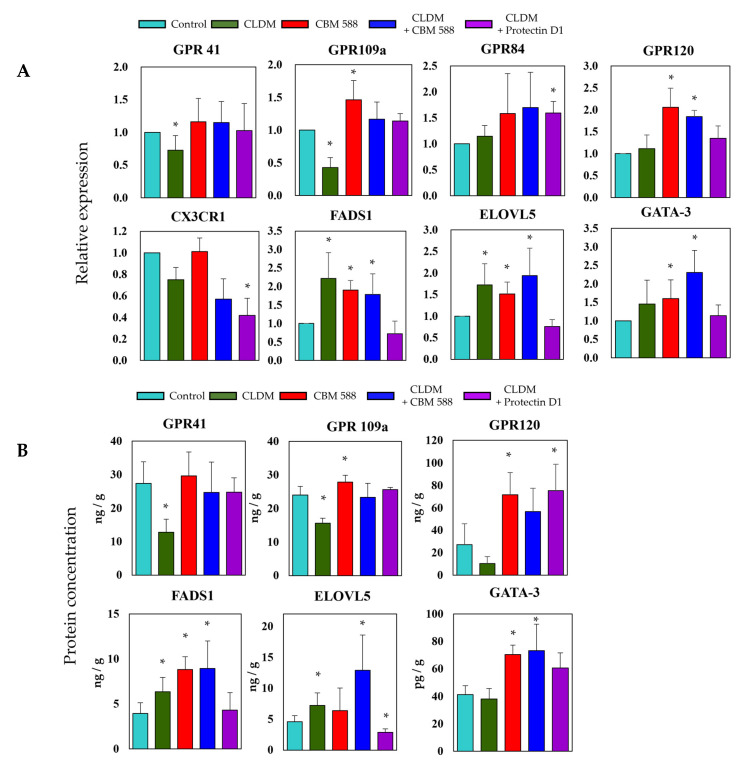
The gut microbe altered by CBM 588 and/or protectin D1 upregulates host fatty acid receptors, lipid elongation enzymes, and immune cell signaling factors. (**A**) Relative RNA levels of genes-encoding enzymes related to fatty acid metabolism and signal factors of immune cells in colon tissue of mice. (**B**) Concentrations of GPR41, GPR109a, GPR120, FADS1, ELOVL5, and GATA-3 in colon tissues of mice. Relative RNA levels of each target gene were normalized to those in the control group (represented as RQ on the vertical axis). Values represent mean ± SD (*n* = 5). Statistical significance was set at *p* < 0.05. * *p* < 0.05, compared with control.

To evaluate the gene expression levels related to fatty acid metabolism and signal factors of immune cells in colon tissue, we evaluated the gene expression levels (Figure 6A) and protein concentration (Figure 6B). The gene expression and protein concentration of short-chain fatty acid receptors 41 and 109a among GPRs were remarkably reduced in the CLDM-alone group. On the other hand, the expression level of GPR120, a long-chain fatty acid receptor, increased in the CBM 588-administered group. The expression of FADS 1 and ELOVL5, which are long-chain fatty acid elongation enzymes, was upregulated in the CLDM-alone administration group and the CBM 588-administration groups. The expression level of GATA-3, which is a factor that differentiates Th0 cells into Th2 cells, was increased in the CBM 588-administered groups.

## 4. Discussion

This trans-omics analysis of the gut microbiome and long-chain fatty acid metabolome in mouse feces was performed to determine the effect of gut microbiota on host lipid metabolism and host-produced metabolites on the gut microbiota.

Gut microbiome analysis revealed that the control group and CBM 588-only administration group had similar gut microbiota (Figure 1B,D). In contrast, CLDM administration reduced the α-diversity of the gut microbiome (Figure 1C). However, the co-administration groups of CBM 588 or protectin D1 with CLDM showed significantly different unweighted UniFrac distances with the CLDM-only administration group (*p* = 0.028), while the CBM 588 + CLDM combination group and protectin D1 + CLDM combination group had similar gut microbiome compositions (*p* = 0.135) (Figure 1D). At the genus level, *Parabacteroides* spp., which are known to cause inflammation with lipopolysaccharide (LPS), increased in the CLDM-only administration group [28]. However, co-administration of CBM 588 or protectin D1 with CLDM increased the abundance of gram-positive bacteria such as *Enterococcus* spp. and *Clostridium* spp. Notably, in the protectin D1 + CLDM administration group, the gut microbiome was similar to that of the CBM 588 + CLDM administration group. *Clostridium* spp. was the most predominant genus, even though CBM 588 was not administered (Figure 1E). Hence, these data suggest that lipid mediators, such as protectin D1, influence the gut microbiome, although this mechanism needs to be elucidated in future studies.

Moreover, functional gene analysis by PICRUSt2 revealed that the gut microbiome in CBM 588 + CLDM and protectin D1 + CLDM combination groups increased functional genes related to unsaturated fatty acids (I: lipid metabolism, III: fatty acid metabolism, IV: biosynthesis of unsaturated fatty acids and V: fatty acid biosynthesis) and/or short-chain fatty acids (II: carbohydrate digestion and absorption) and anti-oxidative stress (VI: ascorbate and aldarate metabolism and VII: glutathione metabolism) (Figure 2). Oxidative stress is often correlated with gut dysbiosis, and it increases inflammatory reactions and subsequent tissue damage [29,30]. Since the oxidative stress-related pathways (VI, VII) containing ascorbic acid and glutathione, which have antioxidant capacity, were significantly enhanced in the CBM 588 + CLDM group (Figure 2), we speculated that CBM 588 changed the gut microbiome to produce antioxidants and protected the host intestinal mucosa. Our previous study revealed that oxidative stress-related biomarkers such as oxidants (malondialdehyde) and antioxidants (total antioxidant capacity colorimetric and superoxide dismutase) were decreased in the CBM588 co-administration group under antibiotic-induced dysbiosis. We speculate that the reason for the decrease in antioxidants is that the anti-inflammatory lipids induced by CBM 588 alleviated inflammation and reduced the causative agents of oxidative stress [9].

On the other hand, alterations in the gut microbiome and its gene functions are linked to the results of metabolomics analysis [3], and some gut bacteria such as *Lactobacillus* spp., *Enterococcus* spp., and *Clostridium* spp. are able to convert linoleic acid to oleic acid via hydroxylated fatty acids, oxo fatty acids, and conjugated fatty acids by a plurality of enzymes [31]. From the results of the target long-chain fatty acid metabolome analysis in feces, 10-hydroxy octadecanoic acid was significantly decreased in the CBM 588 + CLDM combination group and the protectin D1 + CLDM combination group (Figure 3A). Conversely, oleic acid, which is the final transformant of linoleic acid [31], was significantly increased in the CLDM-treated groups. The gut microbiome of the CBM 588 + CLDM and protectin D1 + CLDM administration groups converted linoleic acid to oleic acid more efficiently than the CLDM-only administration group (Figure 3A). As oleic acid has anti-inflammatory and antioxidant properties and has beneficial effects by decreasing both inflammation and oxidative stress [32,33], our data suggested that CBM 588 and protection D1 administration can support anti-inflammatory effects. In addition, our in vitro study with target long-chain fatty acid metabolome analysis in CBM 588 culture broth revealed the opposite results in oleic acid and 10-hydroxy octadecanoic acid concentrations with those of fecal samples. The reduction of linoleic acid and oleic acid, and an increase in 10-hydroxy octadecanoic acid and 10-oxo-12 (Z)-octadecenoic acid were observed in the supernatant of the CBM 588 culture broth (Figure 4B). These results indicate that the CBM 588 strain cannot metabolize linoleic acid to oleic acid. As mentioned above, some *Clostridium* spp. can metabolize linoleic acid to oleic acid via intermediates. In other words, even if they belong to the same genus, some species such as CBM 588 may not have all the enzymes required for conversion from linoleic acid to oleic acid. However, abundant *Lactobacillus* spp., *Enterococcus* spp., and *Clostridium* spp. were confirmed from the results of bacterial flora analysis in the CBM 588-administration group and the protectin D1-administration group (Figure 1E and Figure S1). These results suggest that CBM 588 or protectin D1 administration enhances linoleic acid metabolism by modifying the gut microbiome.

Furthermore, 10-hydroxy-12 (Z)-octadecenoic acid, a CLA, activates the MEK-ERK pathway via GPR40, which is also a receptor for long-chain unsaturated fatty acids and regulates tumor necrosis factor receptor 2 expression in intestinal epithelial cells. As a result, the MEK-ERK pathway via GPR40 suppresses tight junction disorders and inflammation [26]. We previously reported that CBM 588 administration enhances the expression level of GPR120 [9], which is a receptor for long-chain unsaturated fatty acids [34,35]. Since GPR120 is known to react with a ligand common to GPR40, we performed another in vitro study with Caco-2 cells, reported to express GPR120 [27], to investigate which CBM 588 induced-metabolites activate GPR120-mediated signaling. Since GPR120 may induce anti-inflammatory effects as well as GPR40, we measured ERK 1/2 concentration as an index of signal transduction from GPR120, and found that the addition of the supernatant of GAM broth containing linoleic acid after CBM 588 culture significantly increased ERK 1/2 (Figure 5). GPRs activation induces the phosphorylation of ERK 1/2 [26,35,36]. Furthermore, activation of ERK 1/2 induces stabilized ubiquitination of GATA3 and induces the differentiation of naive T cells into Th2 cells [37,38,39]. In our previous study, CBM 588 administration resulted in the upregulation of IL-4 producing Th2 cells [9]. Taken together, GPRs-ERK 1/2-GATA3 signaling cascade, enhanced with CBM 588, can be involved in the differentiation into IL-4 producing Th2 cells [9].

As a result of organic acid measurement in fecal samples, the butyric acid concentration remained comparable to that in the control group in the CBM 588 + CLDM and protectin D1 + CLDM groups. The results indicate that host homeostasis is maintained via the expression of GPR41 and GPR109a, which recognize butyric acid as ligands [34] (Figure 6A,B). Furthermore, in the CBM 588-administration groups, EPA metabolites were significantly induced (Figure 3B). Many studies have shown that fungi and yeasts can produce EPA and its related metabolites [29,30]. However, no data suggest that bacteria can produce EPA and its metabolites, except *Shewanella* spp. and *Colwellia* spp. [40,41]. Hence, no study has revealed that gut bacteria can induce EPA production. On the other hand, having a normal gut microbiome, like SPF mice, was reported to be important for the activation of lipid metabolism-related enzymes such as stearoyl-CoA desaturase 1 and fatty acid elongase 5 in the liver [42]. In our results, the fatty acid elongation-related enzymes FADS1 and ELOVL5 also increased (Figure 6A,B). Hence, we considered that the CBM 588 altered gut microbiome promoted EPA elongation in the host, and EPA metabolites were excreted or transported from the host cells to the lumen side.

Furthermore, CLAs and SCFAs, produced by the gut microbiome, and EPA metabolites produced by the host, react with intestinal epithelial cells [26,43]. Such fatty acid metabolites often function in autocrine and/or paracrine-signaling metabolite target receptors expressed on neighboring cells, such as immune cells [34].

**Figure 7:**
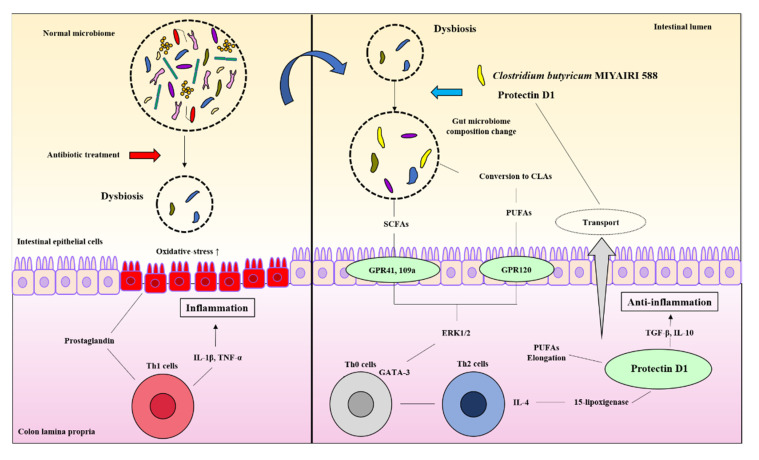
CBM 588 and host-producing lipid mediator modify lipid metabolism by changing the gut microbiome under antibiotic-induced dysbiosis.

Our study revealed some new mechanistic insights into how CBM 588 and host-producing lipid metabolites modulate lipid metabolites via gut microbe alternations against antibiotic-induced gut inflammation (Figure 7). However, as a study limitation, the mouse gut microbiome is not identical to that of the human gut microbiome [44]. Hence, further research is needed to investigate whether these results can be reproduced in humans. Second, female ICR mice of 8–9 weeks have become sexually mature, and the secretion of estrogen may affect the results of the test [45]. However, all mice in each group in this study were under the same conditions to minimize the impact of physiological cycles.

In summary, against antibiotic-induced dysbiosis, administration of LBP and host-produced lipid mediators altered the gut microbiome and exerted an inhibitory effect by increasing anti-inflammatory lipid metabolites in host tissues and feces. To the best of our knowledge, this is the first study to report that oral administration of LBP influences the relationship between colon tissue and gut microbiota in lipid metabolism. These findings provide insights into the mechanisms of prevention and recovery from inflammation and the improvement of host metabolism by CBM 588.

CBM 588 modified the gut microbiome and changed the butyric acid and CLA production levels. Metabolic modifications induce G protein-coupled receptor (GPR) activation in response to oxidative stress and inflammation caused by dysbiosis. The activation of GPR triggers ERK 1/2 signaling and directs the differentiation of downstream immune cells in the host colon tissue. The modified gut microbiome also accelerates the induction of anti-inflammatory lipid mediators (protectin D1) by promoting the elongation of PUFAs in the host. The induced protectin D1 also accelerates gut microbiome modification.

## Data Availability

Not applicable.

## Supplementary Materials

The following are available online at https://www.mdpi.com/article/10.3390/biomedicines9081065/s1, including detailed conditions and experimental methods (targeted long chain fatty acid metabolites analysis using LC-MS/MS, organic acid measurement using HPLC and cell culture and treatment), Figure S1: Bacterial compositions in different experimental groups at the genus level, Figure S2: Heatmaps, generated by hierarchical clustering, of selected functional gene pathway, were significant between each group by Kruskal-Wallis test, Table S1: Linear gradient condition, Table S2: Full MS and data-dependent MS/MS method, Table S3: Ionization method, Table S4. Primers used for quantitative real-time RT-PCR.
