# Supplementary material for: Clostridium butyricum MIYAIRI 588 Modifies Bacterial Composition under Antibiotic-Induced Dysbiosis for the Activation of Interactions via Lipid Metabolism between the Gut Microbiome and the Host
Source: Biomedicines. 2021 Aug 22;9(8):1065. doi: 10.3390/biomedicines9081065 (PMC8391242; doi:10.3390/biomedicines9081065)
Supplement: Supplementary file 1 [file biomedicines-09-01065-s001.zip › Supplemental Figure S1,S2 .pdf]

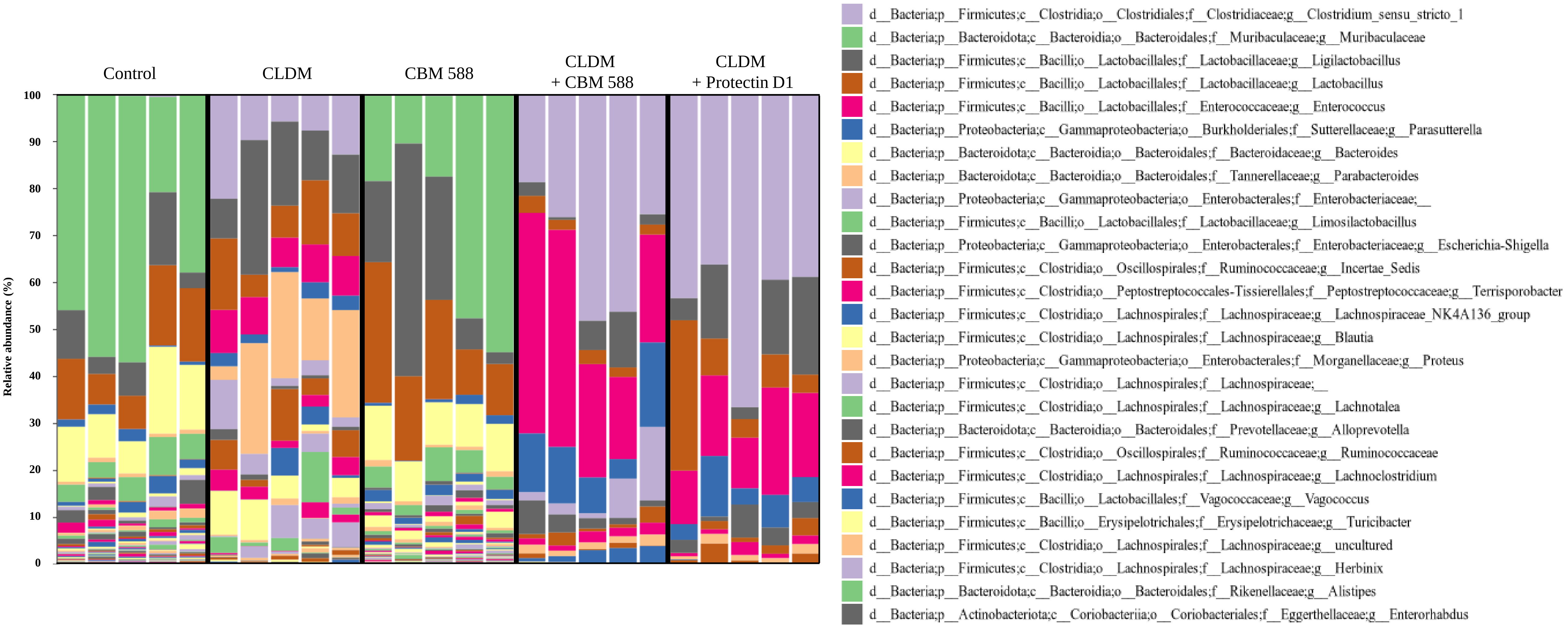

Figure S1. Bacterial compositions in different experimental groups at the genus level. The vertical axis shows relative abundance (%) per the whole (n = 5 in each group).

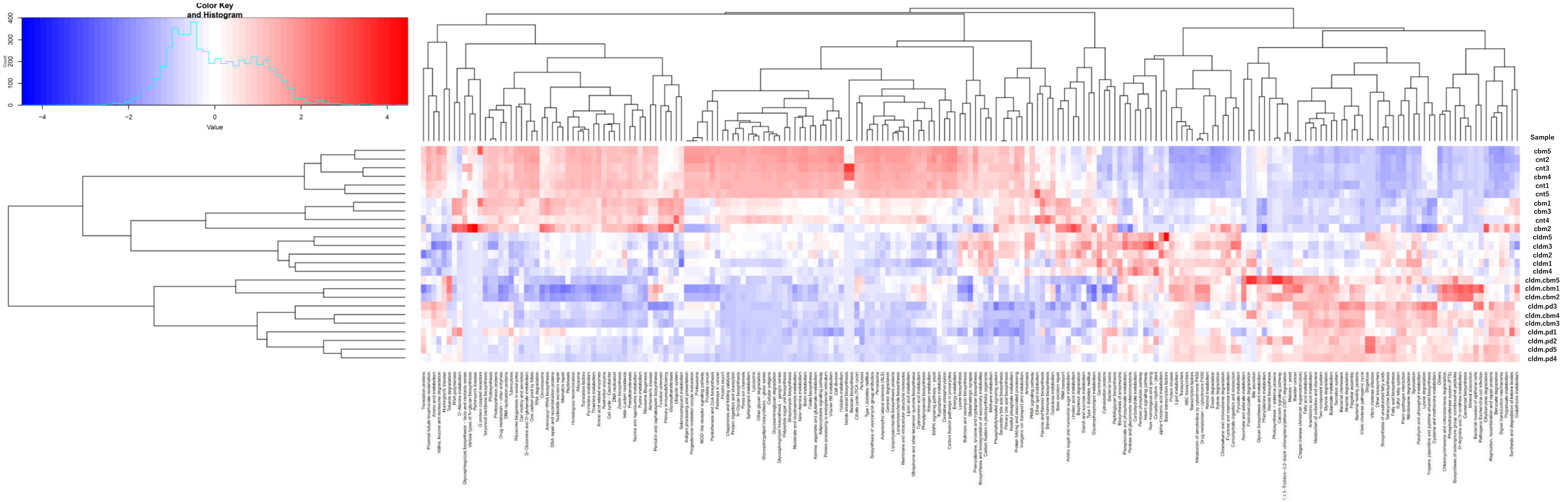

Figure S2. Heatmaps, generated by hierarchical clustering, of selected functional gene pathway, were significant between each group by Kruskal-Wallis test. An FDR *p* value < 0.05 was considered statistically significant, and the Benjamini-Hochberg method was used to calculate FDR *p* value.

The sample name is shown as follows, control group; cnt1-5, CLDM administration group; cldm1-5, CBM 588 administration group; cbm1-5, CBM 588 + CLDM combination group; cldm.cbm1-5 and protectin D1 + CLDM combination group; cldm.pd1-5.
