# Supplementary material for: Clostridium butyricum MIYAIRI 588 Modifies Bacterial Composition under Antibiotic-Induced Dysbiosis for the Activation of Interactions via Lipid Metabolism between the Gut Microbiome and the Host
Source: Biomedicines. 2021 Aug 22;9(8):1065. doi: 10.3390/biomedicines9081065 (PMC8391242; doi:10.3390/biomedicines9081065)
Supplement: Supplementary file 1 [file biomedicines-09-01065-s001.zip › Supplementary Material and Supplemental Tables.pdf]

Supplementary Material

Clostridium Butyricum **MIYAIRI 588 Modifies Bacterial Composition Under Antibiotic-Induced Dysbiosis for the Activation of Interactions Via Lipid Metabolism Between the Gut Microbiome and the Host**

Tadashi Ariyoshi, Mao Hagihara, Susumu Tomono, Shuhei Eguchi, Ayaka Minemura, Daiki Miura, Kentaro Oka, Motomichi Takahashi, Yuka Yamagishi, Hiroshige Mikamo*

Targeted long chain fatty acid metabolites analysis using LC-MS/MS

The orbitrap LC-MS/MS analyses were performed on a Vanquish H system (Thermo Fisher Scientific, OH, USA) using an Acclaim RSLC120 C18 (2.2 μm 2.1 i.d. × 150 mm, Thermo Fisher Scientific) and Q Exactive (Thermo Fisher Scientific) with an electrospray ionization device. The negative ion mode was used. Mobile phase A consisted of MilliQ and 0.1% formic acid (Kanto Chemical Co., Inc., Tokyo, Japan). Acetonitrile (Kanto Chemical Co., Inc.) was used as mobile phase B. Two microliters of the sample was injected and measured. Gradient elution was applied at a flow rate of 400 μL/min using the following time program: 0–0.5 min 20% B, 0.5–15.0 min linear increase to 98% B, 15.0–25.0 min remained at 98% B, 25.0–32.0 min linear decline to 20% B. The linear gradient conditions, MS conditions, and ionization conditions are listed in Tables S1, S2, and S3. The raw data including retention times (Rt), mass-to-charge ratios (m/z), peak area values, MS, and MS/MS fragments were measured in negative ion mode using Xcalibur (Thermo Fisher Scientific) Version 4.1. The following reagents were used as lipid MS/MS library in advance; Primary Prostaglandin Metabolite LC-MS Mixture, Primary Vascular Eicosanoid LC-MS Mixture, Primary COX and LOX LC/MS Mixture, Saturated/Monounsaturated Fatty Acid LC/MS Mixture, SPM D-series LC-MS Mixture, SPM E-series LC-MS Mixture, Oxidative Stress LC-MS Mixture, Polyunsaturated Fatty Acid LC/MS Mixture. These standards were purchased from Cayman, USA. Measured raw data were assigned to lipid metabolites by lipid MS/MS library as mentioned above after normalization, filtering, alignment, and peak identification using Compound Discoverer (Thermo Fisher Scientific) Version 3.1.

Organic acid measurement using high performance liquid chromatography (HPLC)

Feces were sampled from the mouse colon and immediately cryopreserved (stored at -80 °C). For determination of organic acids, 0.1 g of feces was placed in a 2.0 mL tube and homogenized after being suspended in 400 μL of 1× PBS. Samples were vortexed for 1 min, kept on ice for 5 min, and centrifuged at 10,000 × *g* for 5 min at 4 °C. The supernatants were

filtered through a 0.45 μm filter (Merck Millipore, MA, USA). Organic acids (acetic acid, propionic acid, n-butyric acid, iso-valeric acid, succinic acid, and lactic acid) in feces were measured using high-performance liquid chromatography (Prominence, SHIMADZU, Kyoto, Japan). using a post-column reaction with a conductivity detector (CDD-10A, SHIMADZU), two tandemly arranged columns (Shim-pack SCR-102 (H), 300 mm × 8 mm ID, SHIMADZU), and a guard column (Shim-pack SCR-102 (H), 50 mm × 6 mm ID, SHIMADZU). The system was used with a mobile phase (5 mM *p*-toluenesulfonic acid, Kanto Chemical Co., Inc.) and a buffer solution (5 mM *p*-toluenesulfonic acid, 100 μM EDTA, and 20 mM Bis-Tris). The flow rate of the mobile phase and buffer solution were both 0.8 mL/min and the oven temperature was 40 °C. The detector cell temperature was maintained at 48 °C. The measurement sample was contained in 1.0 mL disposable vial (228-31600-91, SHIMADZU). The measurement sample was kept in a sample cooler at 4 °C (SIL-20AC, Shimadzu), and 10 μL was injected using an auto-injector. Succinic acid, lithium DL-lactate, sodium formate, sodium acetate trihydrate, sodium propionate, sodium isobutyrate, n-sodium butyrate, and sodium isovalerate were dissolved in deionized water and adjusted for mixed preparation. Standards other than lithium DL-lactate (FUJIFILM Wako Pure Chemical) were supplied by Kanto Chemical. The retention times of HPLC standards were used to detect the analyzed organic acids on the chromatograms. Moreover, the concentration of acids was calculated using the area under the established peak based on previously prepared standard curves.

Cell culture and treatment

To investigate ligands that highly activate GPR120, which is known to activate MAP kinase ERK 1/2 in different cellular systems, cell activation evaluation by measurement of ERK 1/2 modified from a previous study [26, 27] was conducted as follows. Caco-2 cells were obtained from the American Type Culture Collection (Manassas,VA, USA). The culture medium consisted of Eagle's minimal essential medium (EMEM, FUJI-FILM Wako Pure Chemical Co.) supplemented with 10% fetal bovine serum (Cosmo Bio Co., Ltd.,Tokyo, Japan), 1% nonessential amino acids (Nacalai Tesque, Kyoto, Japan), and antibiotics (100 units / ml penicillin-streptomycin, Thermofisher). Caco-2 cells were cultured at 37 °C in a humidified atmosphere containing 5% CO_2. They were grown in 75 cm^2 tissue culture flasks to 80% confluence and then seeded into a 24-well microplate (AGC Techno Glass Co., Ltd., Shizuoka, Japan) at a density of 2×10^5 cells/cm^2. Caco-2 cells were fed a fresh medium every 48 h. After 14 d of culture, the cells were treated with PUFAs (linoleic acid, α-linolenic acid, eicosapentaenoic acid (EPA), and docosahexaenoic acid (DHA) were purchased form Sigma Aldrich, each 50 μM), GPR120 agonist GW 9508 (Cayman USA Inc.), CBM 588 free culture medium (I: GAM broth and II: GAM broth supplemented with 50 μM linoleic acid, same samples as described in section 2.12), and CBM 588 culture supernatant

(III: supernatant after culturing CBM 588 in GAM broth and IV: supernatant after culturing CBM 588 in GAM broth supplemented with 50 μM linoleic acid, as described in section 2.12). Caco-2 cells were incubated for 48 h. After removing the culture solution and washing with PBS, cells were lysed by adding 500 μL of radioimmunoprecipitation (RIPA) buffer (Nacalai Tesque), and then the cell suspension was collected. After sonication (10 s), the suspension was centrifuged at 10,000 × *g* for 20 min at 4 °C. Protein expression levels of ERK 1/2 (Enzo Life Sciences, MI, USA) in the supernatants were measured using commercially available mouse enzyme-linked immunosorbent assay (ELISA) kits. All procedures were performed according to the manufacturer's instructions.

Table S1. Linear gradient condition.

Min	A (%)	B (%)
0	80	20
0.5	80	20
15	2	98
25	2	98
25	80	20
32	80	20

Table S2. Full MS and data-dependent MS/MS method.

Full MS	Condition
Resolution	7.00×10^4
Scan range (*m*/*z*)	215-800
AGC target	1.00×10^6
Maximum IT (ms)	100
Data-dependent MS/MS	Condition
Resolution	1.75×10^4
Isolation window (*m*/*z*)	±0.8 Da
Stepped normalized collision energy	Step20/40/60

Loop count	3
AGC target	5.00×10^4
Maximum IT (ms)	60
Dynamic exclusion	4 s, 10 s, 30 s
Intensity threshold	8.00×10^4

Table S3. Ionization method.

Parameter	Condition
Ionization	ESI-Negative
Spray voltage (kV)	3
Vaporizer temperature (°C)	350
Capillary temperature (°C)	275
N_2 gas flow rate (Sheath gas)	50
N_2 gas flow rate (Aux gas)	15
S-lens level	50
Probe position	C, +1.75

Table S4. Primers used for quantitative real-time RT-PCR.

Name	Sequence (5′→3′)
GPR41 Forward	GGGGTCGATACAAGAGT
GPR41 Reverse	CTGGCGGAGCTACGTGCT
GPR109a Forward	ATGGCGAGGCATATCTGTGTAGCA
GPR109a Reverse	TCCTGCCTGAGCAGAACAAGATGA
GPR84 Forward	GACTGCCCCTCAAAAGACCTGC
GPR84 Reverse	GCCACGCCCCAGATAATTGC
GPR120 Forward	GTGCCGGGACTGGTCATTGTG
GPR120 Reverse	TTGTTGGGACACTCGGATCTGG
CX3CR1 Forward	CAGCATCGACCGGTACCTT
CX3CR1 Reverse	GCTGCACTGTCCGGTTGTT
FADS1 Forward	GGCTCCCGGGTCATCAG
FADS1 Reverse	ACCCTTGTTGATGTGGAATGC
ELOVL5 Forward	TTCGATGCGTCACTCAGTACCT
ELOVL5 Reverse	TGTCCAGGAGGAACCATCCTT
GATA3 Forward	ACCACAACCACACTCTGGAGGA

GATA3 Reverse	TCGGTTTCTGGTCTGGATGCCT
